# Impacts of subclinical hypocalcemia on physiological, metabolic, and productive responses of Holstein × Gir dairy cows^1^

**DOI:** 10.1093/tas/txaa016

**Published:** 2020-02-04

**Authors:** Rodrigo Rodrigues, Reinaldo F Cooke, Hingryd A O Ferreira, Renato R Florido, Victoria Camargo, Hirys O de Godoy, Giulia A Bruni, José L M Vasconcelos

**Affiliations:** 1 School of Veterinary Medicine and Animal Science, São Paulo State University (UNESP), Botucatu, Brazil; 2 Department of Animal Science, Texas A&M University, College Station, TX

**Keywords:** Holstein × Gir cows, metabolism, milk production, subclinical hypocalcemia

## Abstract

This study compared physiological and productive parameters in ¾ Holstein × ¼ Gir dairy cows diagnosed or not with subclinical hypocalcemia (SCH) during early lactation. Nonlactating, multiparous cows (*n* = 32) were enrolled in this experiment 21 d prior to expected date of calving. Cows were maintained in a single pen with ad libitum access to corn silage before calving and received a limit-fed prepartum concentrate. Cow body weight (BW) and body condition score (BCS) were recorded weekly, and blood samples were collected on days −21, −14, −9, −6, and −3 relative to expected calving. After calving (day 0), cows were managed in a single pen with ad libitum access to a total mixed ration, and were milked twice daily. Cow BW and BCS were recorded upon calving and then weekly. Milk production was recorded daily and milk samples collected weekly until 30 d in milk (DIM). Blood was collected during the first 5 DIM, and at 6, 9, 16, 23, and 30 DIM. Cows were classified with SCH when mean total serum Ca during the first 5 DIM was ≤2.125 mmol/L. Cows diagnosed with SCH (*n* = 11) had less (*P ≤* 0.04) mean BCS (2.85 vs. 3.07; SEM = 0.07) and less concentrations of serum insulin (0.396 vs. 0.738 ppmol/L; SEM = 0.115) and insulin-like growth factor I (35.9 vs. 57.9 ng/mL; SEM = 4.2), and these outcomes were noted since 21 d prior to expected calving. Cows diagnosed with SCH had greater (*P* < 0.01) serum concentrations of cortisol at calving (30.2 vs. 22.4 ng/mL; SEM = 2.0), serum haptoglobin at 3 and 6 DIM (0.453 vs. 0.280 mg/mL on day 3 and 0.352 vs. 0.142 mg/mL on day 6; SEM = 0.046), and tended (*P* = 0.09) to have greater mean concentrations of nonesterified fatty acids from calving to 30 DIM (0.368 vs. 0.304 μEq/L; SEM = 0.026). No differences were detected (*P* ≥ 0.41) for cow BW and milk production. Cows diagnosed with SCH had less (*P* = 0.05) mean concentrations of milk total solids (13.2 vs. 13.8 %; SEM = 0.21), tended to have less (*P* ≤ 0.10) mean concentrations of milk fat (4.34 vs. 4.81 %; SEM = 0.20), protein (3.31 vs. 3.45 %; SEM = 0.05), and lactose (4.45 vs. 4.55 %; SEM = 0.04), and had greater (*P* = 0.02) milk somatic cell count during the initial 14 DIM (504 vs. 140 cells/μL; SEM = 90). Collectively, Holstein × Gir cows diagnosed with SCH upon calving had altered periparturient physiological parameters denoting reduced energy nutritional, increased milk somatic cell count, and less concentration of milk components during early lactation compared with normocalcemic cows.

## INTRODUCTION

Transition dairy cows experience a multitude of physiological challenges associated with parturition and milk synthesis (Grummer, 1995), including hypocalcemia that often depress their immunocompetence and productivity (Goff, 2008). Therefore, nutritional strategies to mitigate hypocalcemia in dairy cattle have been extensively investigated (Santos et al., 2019). Reinhardt et al. (2011) reported that incidence of clinical hypocalcemia in North American dairy herds was relatively low, which can be associated with the inclusion of acidogenic salts in prepartum diets (Santos et al., 2019). However, Reinhardt et al. (2011) also reported that prevalence of subclinical hypocalcemia (SCH) was noteworthy, being 25% for primiparous and 47% for multiparous cows.

The impacts of hypocalcemia on dairy cattle welfare and production extend beyond its clinical symptoms. Inadequate concentrations of ionized Ca in the circulation impaired immune cell function and smooth muscle contraction (Hansen et al., 2003; Kimura et al., 2006). Accordingly, SCH may increase susceptibility to retained placenta, mastitis, and ketosis (Reinhardt et al., 2011), reduce feed intake and rumen function (Goff, 2008), and affect inflammatory responses and energy status in transition cows (Martinez et al., 2012). Therefore, current research has focused on causes for SCH incidence, and also development of strategies to mitigate this syndrome in dairy cattle (Neves et al., 2017).

The vast majority of hypocalcemia-based research, however, has been conducted with high-producing Holstein cows (Santos et al., 2019). Studies investigating hypocalcemia in dairy cattle from tropical regions of the planet is extremely limited, including Holstein × Gir cows that have reduced milk yield compared with Holstein cattle (Madalena et al., 1979). Therefore, this experiment compared physiological, metabolic, and productive parameters during the transition period between Holstein × Gir dairy cows classified as SCH or not upon parturition. We hypothesized that SCH is also relevant to transition Holstein × Gir dairy cows, whereas SCH impairs the metabolic and physiological responses that regulate their welfare and productivity.

## MATERIALS AND METHODS

This experiment was conducted at the São Paulo State University—Lageado Experimental Station, located in Botucatu, São Paulo, Brazil. Experimental protocol was reviewed and approved by the São Paulo State University—Animal Ethics Committee (#108/2018).

### Animals and Diets

Cows evaluated herein were part of a companion project focused on supplementation acidogenic salts prior to calving, and fully described by Rodrigues et al. (2018). Pertaining to the current experiment, 32 nonlactating pregnant ¾ Holstein × ¼ Gir cows in their second (*n* = 20), third (*n* = 8), and fourth (*n* = 4) lactation [mean ± SE; initial body weight (BW) = 611 ± 14 kg, initial body condition score (BCS) = 3.25 ± 0.06, 2.5 ± 0.1 parities] were assigned to experimental procedures 21 d prior to expected date of calving. Cows were maintained in a single drylot pen with ad libitum access to corn silage and water, and individually received a limit-fed prepartum concentrate through self-locking head gates once daily (0800 h). After calving, cows were moved to an adjacent single drylot pen with ad libitum access to water and a total mixed ration. Composition and nutritive values of prepartum concentrate and lactation total mixed ration are described by Rodrigues et al. (2018) and summarized in Table 1. Cows were milked twice daily in a side-by-side milking system (0600 and 1700 h), and the experiment was terminated when cows reached 30 d in milk (DIM). The total mixed ration was formulated to yield 35 kg of milk/d using the Spartan Dairy Ration Evaluator/Balancer (version 3.0; Michigan State University, East Lansing, MI).

**Table 1.** Composition and nutritional profile of prepartum concentrate or lactation total mixed ration offered to ¾ Holstein × ¼ Gir dairy cows

Item	Prepartum	Lactation
Composition, % as dry matter basis		
Corn silage	—	47.8
Ground corn	33.0	26.1
Soybean meal	38.7	23.2
Limestone	10.7	—
Sodium chloride	0.650	—
Dicalcium phosphate	3.83	—
Prepartum mineral mix^a^	13.1	—
Lactation mineral mix^a^	—	2.61
Urea	—	0.260
Total intake, kg/d as dry matter basis	2.91	Ad libitum
Nutritional value,^b^ dry matter basis		
Dry matter, %	90.5	44.3
Neutral detergent fiber, %	10.0	28.7
Metabolizable energy, Mcal/kg	2.71	2.58
Crude protein, %	24.4	19.0
Ca, %	5.81	0.80
P, %	1.18	0.56
Mg, %	0.74	0.27
Cl, %	1.98	0.25
K, %	1.05	1.18
Na, %	0.415	0.22
S, %	0.805	0.23
Co, mg/kg	2.69	0.13
Cu, mg/kg	45.9	19.0
I, mg/kg	1.53	0.65
Fe, mg/kg	1,024	101
Mn, mg/kg	165	38.7
Se, mg/kg	0.795	0.46
Zn, mg/kg	173	70.6
Vitamin A, IU/kg	38,350	5,200
Vitamin D, IU/kg	9,600	1,300
Vitamin E, IU/kg	383	26.1
Dietary cation–anion difference,^c^ mEQ/100 g	−61.3	20.1

^a^Composition of supplements described by Rodrigues et al. (2018).

^b^Based on nutritional composition of concentrate ingredients analyzed via wet chemistry procedures by a commercial laboratory (3rlab, Belo Horizonte, Brazil).

^c^Dietary cation–anion difference = [(Na % of DM/0.023) + (K % of DM/0.039)] − [(S % of DM/0.016) + (Cl % of DM/0.0355)].

### Sampling

Throughout the experiment, cows were monitored daily by 2 trained research technicians for incidence of health disorders, including retained placenta, lameness, ketosis, mastitis, and metritis (Rodrigues et al., 2018). Cows were observed for clinical signs of hypocalcemia including hypersensitivity and excitability (stage 1), inability to stand but maintenance of sternal recumbency (stage 2), and loss of consciousness (stage 3). No clinical health disorders including clinical hypocalcemia were detected during the experiment.

Prior to calving, cow BW and BCS were scheduled to be recorded once weekly (days −21, −14, and -7) and blood collected on days −21, −14, −9, −6, and −3 relative to expected calving date (day 0). All samplings were performed prior to concentrate feeding (0800 h) of the day. Based on actual calving dates, cows were enrolled in this experiment on day 19.1 ± 0.8 prior to calving. Hence, day of BW, BCS, and blood collections relative to actual calving date were rounded into the nearest prescheduled sampling date.

Cow BW and BCS were recorded immediately after calving (day 0) and then once weekly until 30 DIM. Blood samples were collected prior to each milking during the first 5 DIM (0600 and 1700 h), as well as at 6, 9, 16, 23, and 30 DIM prior to the morning milking (0600 h). Individual milk production was recorded daily until 30 DIM, whereas milk samples were collected once a week from each cow following each milking of the day. More specifically, 50 mL were retrieved from a composite milk sampler (#AMS/200; Ambic Equipment Limited, Oxfordshire, UK) attached to each individual milk collector (GEA Farm Technologies, Bönen, Germany), mixed with a bronopol preservative, and stored at 4 °C.

### Laboratorial Analysis

Samples from both milkings of the day were combined into 1 daily sample (100 mL) and shipped to a commercial laboratory (Clínica do Leite; Universidade de São Paulo, Piracicaba, Brazil). Milk samples were analyzed for somatic cell count (SCC) and concentrations of fat, lactose, protein, and total solids as described by Rodrigues et al. (2018). Daily milk yield was adjusted to fat-correct milk (FCM), energy-corrected milk (ECM), or total solids (TS)-corrected milk (National Research Council [NRC], 2001) based on milk concentrations of fat, protein, and total solids of the concurrent week.

Blood samples were collected from either the coccygeal vein or artery into commercial blood collection tubes (Vacutainer, 10 mL; Becton Dickinson, Franklin Lakes, NJ). After collection, blood samples were placed immediately on ice, allowed to clot for 24 h at 4 °C, centrifuged at 3,000 × *g* at room temperature for 15 min for serum collection, and stored at −20 °C. Blood samples collected during the first 5 DIM were analyzed for total serum Ca concentration (colorimetric kit #K051; Bioclin Diagnostics, Belo Horizonte, Brazil). Cows were classified with SCH when mean total serum Ca during the initial 5 DIM was ≤2.125 mmol/L and normocalcemic (NORM) when >2.125 mmol/L (Martinez et al., 2016). Blood samples collected pre-calving (−21, −14, −9, −6, and −3 relative to expected calving date) and post-calving (0, 3, 6, 9, 16, 23, and 30 DIM) were analyzed for serum concentrations of glucose, nonesterified fatty acids (NEFA), haptoglobin, insulin-like growth factor (IGF)-I, cortisol, and insulin as described by as described by Rodrigues et al. (2018). The intra- and interassay CV were, respectively, 2.9% and 3.9% for glucose, 3.8% and 5.3% for NEFA, 3.0% and 8.7% for haptoglobin, 4.8% and 6.3% for cortisol, and 4.0% and 5.0% for insulin. Serum IGF-I concentration was analyzed within a single assay, and the intra-assay CV was 2.7%.

### Statistical Analysis

Cow was considered the experimental unit for all analyses. All data were initially tested for normality with the Shapiro–Wilk test from the UNIVARIATE procedure (SAS Inst., Inc., Cary, NC), and only milk SCC data were not normally distributed (*W* = 0.41). Hence, SCC data were log transformed (base-10 log) to achieve normality (*W* = 0.96) for statistical analysis, and results back transformed to facilitate interpretation. All data were analyzed with the MIXED procedure of SAS (SAS Inst. Inc.). All model statements contained the fixed effects of calcemia (SCH or NORM), parity (second, third, or fourth lactation), time (hour, day, or week), and the resultant interaction. Cow (calcemia × parity) was included as random variable in all models. The specified term for the repeated statements varied according to response (hour, day, or week) and cow (calcemia × parity) was included as subject. The covariance structure utilized for all analyses was autoregressive, which yielded the lowest Akaike information criterion value. Previous 305-d mature-equivalent milk yield from each cow was also included as independent covariate for all analyses of daily milk production and composition. Significance was set at *P* ≤ 0.05 and tendencies were determined if *P* > 0.05 and ≤ 0.10. Results are reported as least square means, or covariately adjusted if related to milk production and composition, and separated using least square differences. Repeated measures are reported in the text according to main calcemia effect, or according to the highest-order interaction containing the calcemia effect with *P* ≤ 0.10.

## RESULTS

**Figure 1.**
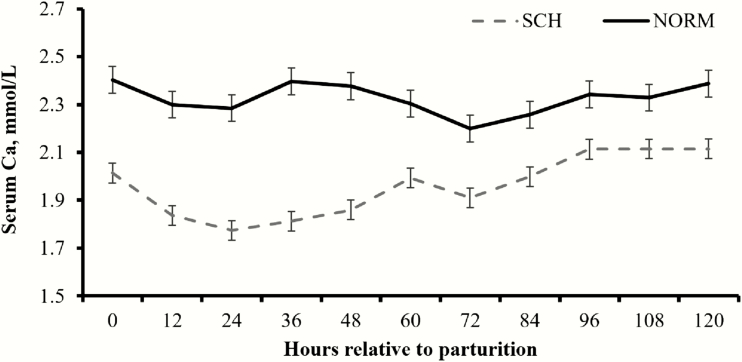
Serum concentrations of total Ca in ¾ Holstein × ¼ Gir dairy cows during the transition period according to calcemia. Calved occurred on day 0, and blood samples were collected twice daily during the initial 5 d in milk. Cows were classified with subclinical hypocalcemia (SCH, *n* = 11) when mean total serum Ca was ≤2.125 mmol/L and normocalcemic (NORM, *n* = 21) when >2.125 mmol/L. Cows classified as NORM had greater (*P* < 0.01) mean concentration of total serum Ca compared with SCH cows. No calcemia × hour interaction was noted (*P* > 0.51). Results are reported as least square means ± SEM.

**Figure 2.**
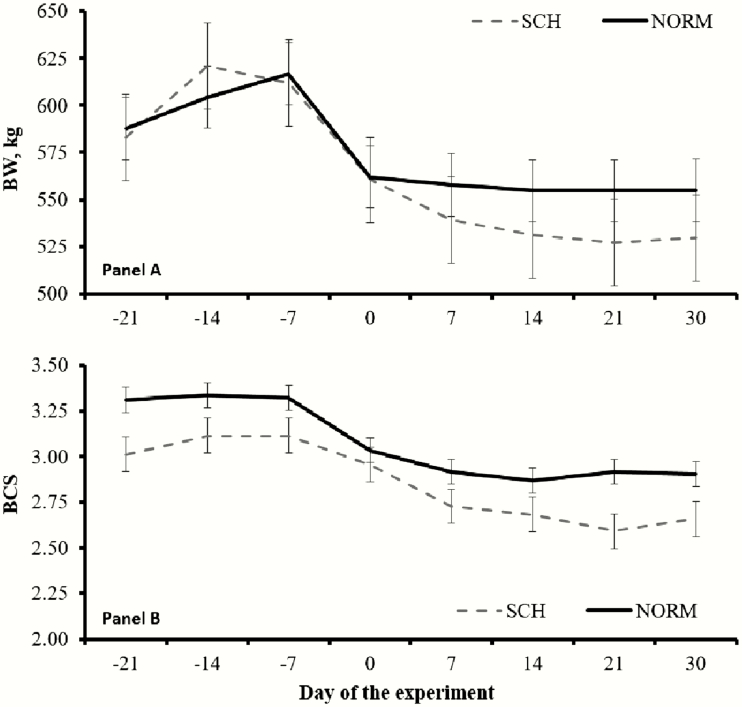
Body weight (BW; A) and body condition score (BCS; B) in ¾ Holstein × ¼ Gir dairy cows during the transition period according to calcemia. Calved occurred on day 0, and blood samples were collected twice daily during the initial 5 d in milk. Cows were classified with subclinical hypocalcemia (SCH, *n* = 11) when mean total serum Ca was ≤2.125 mmol/L and normocalcemic (NORM, *n* = 21) when >2.125 mmol/L. No BW differences were noted (*P* > 0.68) between NORM and SCH cows, whereas mean BCS was less (*P* < 0.01) in SCH compared with NORM cows. No calcemia × day interactions were noted for both variables (*P* ≥ 0.21). Results are reported as least square means ± SEM.

The interaction of calcemia (SCH or NORM) with the treatments imposed by Rodrigues et al. (2018) was tested across all variables investigated, and no significant interactions were observed (*P* > 0.37). Hence, results reported herein were independent of the treatments from Rodrigues et al. (2018). Overall, 11 cows were classified as SCH, whereas 21 as NORM, resulting in an SCH incidence of 34.3%. As designed, NORM cows had greater (*P* < 0.01; Fig. 1) mean concentration of total serum Ca throughout the initial 5 DIM compared with SCH cows (2.325 vs. 1.962 mmol/L, respectively; SEM = 0.026). No differences in mean BW were noted (*P* = 0.68; Fig. 2a) between SCH and NORM cows (563 vs. 574 kg, respectively; SEM = 18). Mean BCS during the experiment was less (*P* < 0.01) in SCH compared with NORM cows (2.85 vs. 3.07, respectively; SEM = 0.07), and such outcome was noted since the beginning of the experiment (Fig. 2b). Day effects were detected (*P* < 0.01) for BW and BCS, as both decreased upon parturition across calcemia groups (Fig. 2).

**Figure 3.**
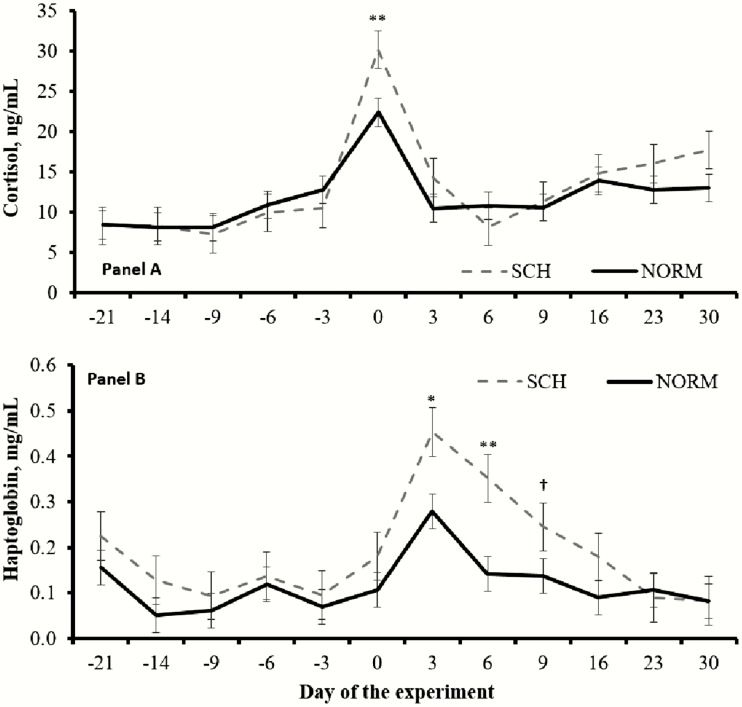
Serum concentrations of cortisol (A) and haptoglobin (B) in ¾ Holstein × ¼ Gir dairy cows during the transition period according to calcemia. Calved occurred on day 0, and blood samples were collected twice daily during the initial 5 d in milk. Cows were classified with subclinical hypocalcemia (SCH, *n* = 11) when mean total serum Ca was ≤2.125 mmol/L and normocalcemic (NORM, *n* = 21) when >2.125 mmol/L. Calcemia × day interactions were detected (*P* ≤ 0.05) for both responses. Within days; **P < 0.01, *P ≤ 0.05, and ^†^*P* ≤ 0.10. Results are reported as least square means ± SEM and separated using least square differences.

**Figure 4.**
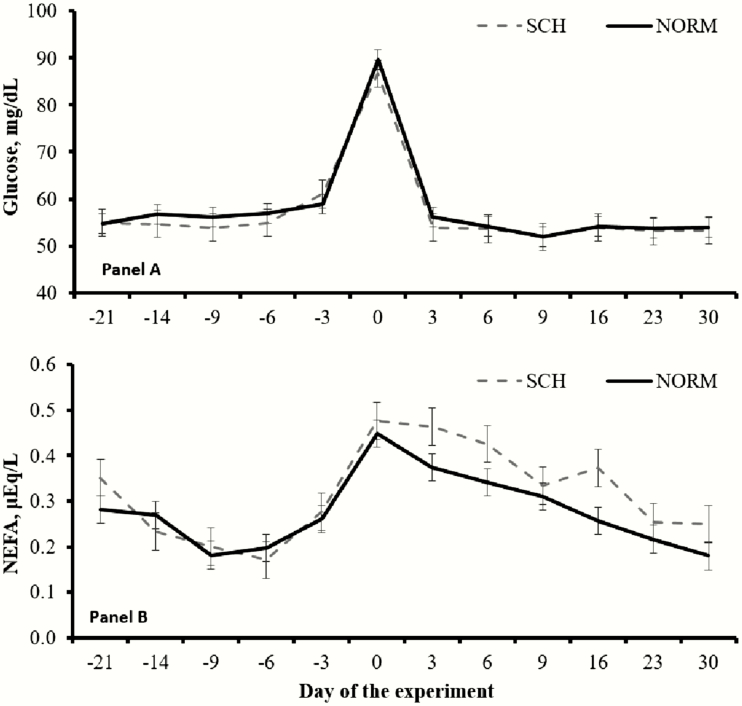
Serum concentrations of glucose (A) and nonesterified fatty acids (NEFA; B) in ¾ Holstein × ¼ Gir dairy cows during the transition period according to calcemia. Calved occurred on day 0, and blood samples were collected twice daily during the initial 5 d in milk. Cows were classified with subclinical hypocalcemia (SCH, *n* = 11) when mean total serum Ca was ≤2.125 mmol/L and normocalcemic (NORM, *n* = 21) when >2.125 mmol/L. No differences between SCH and NORM cows nor calcemia × day interactions were detected (*P* ≥ 0.15) for both variables. Results are reported as least square means ± SEM.

Calcemia × day interactions were detected (*P* ≤ 0.05) for serum cortisol and haptoglobin concentrations. Immediately after calving, serum cortisol was greater (*P* < 0.01) in SCH compared with NORM cows (Fig. 3a). Serum haptoglobin concentrations were also greater (*P* < 0.01) at 3 and 6 DIM and tended to be greater (*P* = 0.10) at 9 DIM in SCH compared with NORM cows (Fig. 3b). Day effects were detected (*P* < 0.01) for serum cortisol and haptoglobin, as both increased upon parturition across calcemia groups (Fig. 3). No differences in mean serum glucose concentrations were noted (*P* = 0.52) between SCH and NORM cows during the experiment (57.1 vs. 58.1 mg/dL, respectively; SEM = 1.0), despite a day effect (*P* < 0.01) as serum glucose peaked immediately after calving (Fig. 4a). Mean serum concentrations of NEFA also did not differ (*P* = 0.15) between SCH and NORM cows (0.317 vs. 0.276 μEq/L, respectively; SEM = 0.019) and increased for both calcemia groups after calving (day effect, *P* < 0.01; Fig. 4b). Nevertheless, mean concentrations of serum NEFA during the postpartum period only (0 to 30 DIM) tended to be greater (*P* = 0.09) in SCH compared with NORM cows (0.368 vs. 0.304 μEq/L, respectively; SEM = 0.026).

**Figure 5.**
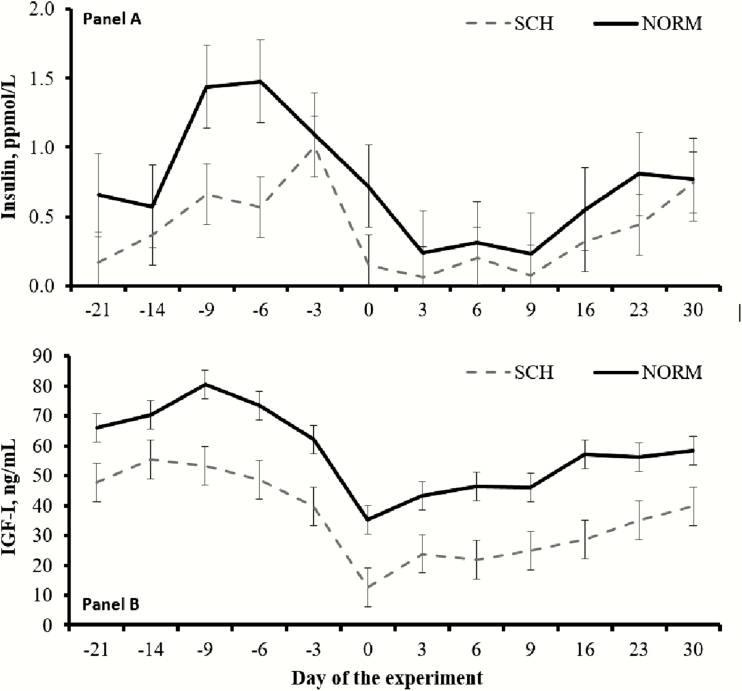
Serum concentrations of insulin (Panel A) and insulin-like growth factor (IGF)-I (Panel B) in ¾ Holstein × ¼ Gir dairy cows during the transition period according to calcemia. Calved occurred on day 0, and blood samples were collected twice daily during the initial 5 d in milk. Cows were classified with subclinical hypocalcemia (SCH, *n* = 11) when mean total serum Ca was ≤2.125 mmol/L, and normocalcemic (NORM, *n* = 21) when >2.125 mmol/L. Cows classified as NORM had greater (*P* ≤ 0.04) mean concentrations of insulin and IGF-I compared with SCH cows. No calcemia × day interactions were noted for both variables (*P* ≥ 0.71). Results are reported as least square means ± SEM.

Cows classified as SCH had less (*P* ≤ 0.04) mean concentrations of insulin and IGF-I compared with NORM cows (0.396 vs. 0.738 ppmol/L for insulin, SEM = 0.115; 35.9 vs. 57.9 ng/mL for IGF-I, SEM = 4.2, respectively), and such outcomes were also noted since the beginning of the experiment (Fig. 5). Day effects were detected (*P* < 0.01) for both hormones, as these decreased as parturition approached (Fig. 5). No differences were detected (*P* ≥ 0.60) for milk production parameters between SCH and NORM cows, including fat-corrected milk, total solids-corrected milk, and energy-corrected milk (Table 2). In turn, SCH cows had less (*P* = 0.05) mean concentrations of milk total solids and tended (*P* ≤ 0.10) to have less mean concentrations of milk fat, protein, and lactose compared with NORM cows (Table 2). A calcemia × day interaction (*P* = 0.04) was detected for milk SCC, which was greater (*P* ≤ 0.05) in SCH compared with NORM cows during the initial 2 wk of lactation (442 vs. 171 cells/μL in week 1 and 576 vs. 115 cells/μL in week 2, respectively; SEM = 117), but did not differ (*P* ≥ 0.19) afterwards (401 vs. 185 cells/μL in week 3 and 175 vs. 321 cells/μL in week 4, respectively; SEM = 117).

**Table 2.** Milk production during the first 30 d in milk in ¾ Holstein × ¼ Gir dairy cows classified with subclinical hypocalcemia (SCH; *n* = 11) or normocalcemia (NORM; *n* = 21)^a,b,c^

Item	SCH	CON	SEM	*P*-value
Milk production				
Milk yield, kg/d	24.3	23.9	1.2	0.81
Fat-corrected milk, kg/d	27.7	28.8	1.5	0.60
12% total solids-corrected milk, kg/d	26.9	27.5	1.4	0.78
Energy-corrected milk, kg/d	27.9	29.0	1.5	0.62
Milk composition				
Fat, %	4.34	4.81	0.20	0.09
Protein, %	3.31	3.45	0.05	0.10
Lactose, %	4.45	4.55	0.04	0.06
Total solids, %	13.23	13.84	0.21	0.05
Somatic cell count, cells/μL	398	198	74	0.05

^a^Results are reported as least square means covariately adjusted to previous 305-d mature-equivalent milk yield from each cow, and separated using least square differences.

^b^Blood samples were collected twice daily during the initial 5 DIM. Cows were classified as SCH when mean total serum Ca was ≤2.125 mmol/L and as NORM when >2.125 mmol/L.

^c^Fat corrected, energy corrected, and total solids corrected were calculated based on daily milk yield and concentrations of fat, protein, and total solids of the concurrent week (NRC, 2001).

## DISCUSSION

Cows evaluated in this experiment experienced the physiological challenges associated with calving and beginning of lactation, as evidenced by temporal changes in BW, BCS, and all serum metabolites and hormones (Jorritsma et al., 2003). However, clinical disorders typical of the transition period were not observed in SCH and NORM cows, including clinical hypocalcemia, clinical mastitis, and retained placenta. This latter outcome can be associated with the use of acidogenic salts in prepartum diets, corroborating a recent meta-analysis by Santos et al. (2019). Lack of clinical hypocalcemia can also be related to the young parity of cows (Rezac et al., 2014), and the moderate milk yield and consequent Ca demand for milk synthesis of Holstein × Gir cattle (Madalena et al., 1979). The 34.3% of cows classified as SCH herein was within the range reported by Reinhardt et al. (2011) in North American Holstein-based dairies and demonstrates that this syndrome is also relevant to Holstein × Gir dairy cows. It should be noted that Reinhardt et al. (2011) used a lower serum Ca threshold from a single blood sample to evaluate SCH prevalence (<2.0 mmol/L threshold). Yet, the criteria adopted for SCH herein was the same used in the companion project (Rodrigues et al., 2018), and based on previous research that collected multiple blood samples during the initial 4 DIM (Martinez et al., 2016). Nonetheless, mean concentration of total serum Ca in SCH cows was below 2.0 mmol/L and nearly 20% less compared with that of NORM cows; a relevant difference considering the precise homeostatic control of circulating Ca in dairy cattle (Horst et al., 1986). Serum samples collected prior to calving were not analyzed for total Ca concentration, given the current hypothesis was based on serum Ca during the first 5 DIM. Future research should also consider the association between prepartum serum Ca status and SCH incidence in Holstein × Gir dairy cows, given that circulating concentration of total Ca prior to calving (≤2.4 mmol/L threshold) has been identified as a predictor for SCH in Holstein cattle (Neves et al., 2017).

Body condition score is the most common visual indicator of energy reserves (Wildman et al., 1982), whereas serum insulin and IGF-I concentrations often reflect nutrient status in dairy cattle (Butler, 2000). According to differences noted between calcemia groups on BCS, serum insulin and IGF-I, energy status during prepartum was reduced in SCH compared with NORM cows, which persisted throughout the experimental period. Lack of similar outcomes in cow BW and serum glucose can be associated, respectively, with the contribution of gastrointestinal tract content to BW variability and rigorous homeostatic regulation of circulating glucose in ruminants (Bickerstaffe et al., 1974). Others have also reported that SCH worsens the negative energy balance of early-lactating dairy cattle, including greater circulating NEFA and reduced insulin concentrations (Reinhardt et al., 2011; Martinez et al., 2014). Besides reducing voluntary feed intake, SCH directly hinders pancreatic insulin release by limiting Ca influx into pancreatic cells, which in turn stimulates lipid mobilization via hormone-sensitive lipase (Martinez et al., 2014). Accordingly, SCH cows had increased serum NEFA concentrations during the postpartum period herein, also denoting greater lipolysis to support milk yield despite nutrient shortage compared with NORM cows (Grummer, 1995). Results from this experiment also suggest energy status as a predisposing factor for SCH in transition Holstein × Gir dairy cows. To our knowledge, no other research has investigated BCS, insulin, and IGF-I as predictors of SCH in dairy cattle, particularly cows with *Bos indicus* influence. Yet, negative nutrient balance prepartum has been speculated to precede or exacerbate circulating Ca insufficiency (Neves et al., 2017), whereas SCH during early lactating may be symptomatic of inadequate prepartum nutrient intake (Seifi et al., 2011).

Previous research reported that SCH resulted in increased plasma cortisol concentrations within 36 h postpartum (Horst and Jorgensen, 1982) and altered innate immunocompetence of early-lactating dairy cows (Kimura et al., 2006; Martinez et al., 2012). Accordingly, SCH cows herein had greater serum cortisol concentrations immediately after calving, and such outcome disappeared 72 h later. Moreover, SCH cows had greater serum haptoglobin concentrations during the initial 9 DIM. Haptoglobin is a proinflammatory acute-phase protein often used as a biomarker for diseases in dairy cattle (Murata et al., 2004) and associated with elevated cortisol concentrations (Cooke, 2017). Despite the lack of clinical disorders, these outcomes corroborate that SCH intensified the corticosteroid response elicited by metabolic stress and trauma from parturition, and heightened the acute-phase protein reaction typical of early-lactating dairy cattle (Hudson et al., 1976; Rodrigues et al., 2018). These responses may be associated with a metabolic attempt of the organism to recover from hypocalcemia and restore homeostasis, particularly due to the hypophosphatemic effects of cortisol (Horst and Jorgensen, 1982).

Haptoglobin also serves as biomarker for inflammatory processes such as subclinical mastitis (Murata et al., 2004), which results in elevated milk SCC (Ruegg, 2003). Accordingly, milk SCC was greater in SCH compared with NORM cows during the initial 2 wk of lactation, concurrently with serum haptoglobin differences between calcemia groups. Earlier research already established that the immunosuppression caused by SCH decreases the ability of lactating cows in controlling infections, including in the mammary gland (Kimura et al., 2006). In turn, studies investigating the impacts of SCH on milk composition are limited. Cows classified with SCH herein had reduced milk TS compared with NORM cows, resultant from less milk concentrations of fat, protein, and lactose. Chamberlin et al. (2013) also reported less milk protein during the initial 35 DIM in cows classified with SCH, despite similar milk fat and total solids compared with normocalcemic cohorts. The exact reasons for these outcomes are still unknown, but may be related to inferior energy status of SCH during early lactation. Indeed, nutritional status modulates biosynthesis of milk fat, protein, and lactose and is positively associated with milk content of these nutrients in dairy cattle (Osorio et al., 2016). The improved energy balance of NORM cows, however, did not result in increased milk production compared with SCH cows, including FCM, ECM, and TS-corrected milk (Akers, 2006). Alternatively, Martinez et al. (2012) proposed that cows with SCH are more efficient in milk production and perhaps capable of maintaining overall productivity similar to NORM cows despite reduced nutrient availability.

In summary, this study provides novel information about SCH in transition Holstein × Gir dairy cows, although results presented herein do not establish cause and effect mechanisms. Within the population evaluated (*n* = 32), 34.3% of cows were classified with SCH upon calving, whereas their energy status was inferior during the prepartum period and remained inferior during early lactation compared with NORM cows. Cows classified with SCH also had heightened serum cortisol and haptoglobin responses to calving and onset of lactation, which was accompanied by increased milk SCC during the initial 14 DIM. Milk solid composition during early lactation was less in SCH cows, although overall milk production was not altered by SCH classification. Collectively, these outcomes support the conclusion that SCH is relevant to Holstein × Gir dairy cows, and appear to be associated with prepartum nutrient reserves. Therefore, strategies to mitigate this syndrome are warranted to promote welfare and production of Holstein × Gir cattle, including dietary management to ensure adequate nutritional status during the transition period.
